# Key periodontal pathogens may mediate potential pathogenic relationships between periodontitis and crohn’s disease

**DOI:** 10.1186/s12903-024-04425-0

**Published:** 2024-06-07

**Authors:** Boyang Sun, Ying Wang, Mengmeng Wu, Geng Feng, Ting Guo

**Affiliations:** 1 Department of General Dentistry, Research institute of Stomatology, Nanjing stomatological Hospital, Affiliated hospital of medical school, Nanjing University, Nanjing, 210008 China; 2 Department of Pharmacy, Research institute of Stomatology, Nanjing stomatological Hospital, Affiliated hospital of medical school, Nanjing University, Nanjing, 210008 China; 3 Nanjing Fengzi Bio-pharm Technology Co. Ltd, Nanjing, 210018 China

**Keywords:** Crohn’s disease, Periodontitis, Periodontal pathogens, *Porphyromonas gingivalis*, *Fusobacterium nucleatum*

## Abstract

**Background:**

Crohn’s disease (CD)-associated periodontitis is common. However, the role of periodontal pathogens in the Coexistence of CD and periodontal disease remains unclear.

**Methods:**

To investigate the potential relationship mediated by periodontal pathogens between periodontitis and CD, we collected salivary samples from healthy participants (H group, *n* = 12), patients with CD (Ch group, *n* = 10), patients with periodontitis (Ps group, *n* = 12), and patients with Coexistence of CD and periodontal disease (Cp group, *n* = 12) and analyzed them by 16 S rRNA sequencing.

**Results:**

Patients with Coexistence of CD and periodontal disease had increased levels of *Fusobacterium*, *Actinomyces*, *Leptotrichia*, and *Prevotella*, which correlated with the severity of periodontitis. Conversely, the levels of *Streptococcus*, *Neisseria*, *Haemophilus*, and *Gemella*, which decreased in Coexistence of CD and periodontal disease, were negatively correlated with the severity of periodontitis. To further investigate the role of periodontal pathogens in CD development, representative periodontal pathogens causing periodontitis, *Porphyromonas gingivalis* and *Fusobacterium nucleatum*, were administered to mice. These pathogens migrate to, and colonize, the gut, accelerating CD progression and aggravating colitis, and even systemic inflammation. In vitro experiments using a Caco-2/periodontal pathogen coculture revealed that *P. gingivalis* and *F. nucleatum* increased intestinal permeability by directly disrupting the tight junctions of intestinal epithelial cells.

**Conclusion:**

Our findings strongly suggest that periodontal pathogens play a role in the relationship between periodontitis and CD. These results provide a basis for understanding the pathogenesis of Coexistence of CD and periodontal disease and may lead to the development of novel therapeutic strategies.

**Supplementary Information:**

The online version contains supplementary material available at 10.1186/s12903-024-04425-0.

## Background

Crohn’s disease (CD) is a chronic inflammatory bowel disease (IBD) and a major subtype of IBD. Its incidence is increasing worldwide [1]. IBD is a systemic disease not limited to the gastrointestinal tract, and oral lesions may be the main extraintestinal manifestations [2]. Among all the oral niches, the periodontium is the key to the oral system link [3]. Several studies have reported a significantly higher prevalence of periodontitis in patients with CD than in healthy controls [4–6]. A meta-analysis demonstrated that CD is associated with an increased risk of periodontitis [7]. Moreover, it is reported that the induction of periodontitis results in gut dysbiosis and altered gut epithelial cell barrier function [8, 9]. In essence, IBD and periodontitis affect each other’s progression through a bidirectional relationship [10, 11].

Differences in oral microbial communities have been reported, which may be partly due to specific characteristics of patients [12–15]. For example, IBD patients harbor more bacteria associated with opportunistic infections than subjects without IBD [12]. In addition, the gut microbiome of patients with IBD is significantly more similar to the oral microbiome compared with that of IBD-free controls [15]. Several mechanisms have been proposed to explain the effects of periodontal disease on intestinal inflammation. These connections include: (1) systemic dissemination of periodontal pathogens and inflammatory mediators through the bloodstream [16]; (2) dysbiosis caused by the ectopic colonization of oral bacteria in the intestine, leading to disruption of the gut microbiota [9]; and (3) the gum–gut axis, which appears to be inherently connected through saliva, which serves as a conduit for the transmission of enzymes, effector cytokines, bacteria, and viable subsets of inflammatory cells to the intestine [17]. However, the exact mechanisms underlying the interactions between oral and intestinal diseases remain unclear. The direction and causality of these associations are also uncertain. Therefore, in this study, saliva samples from patients with CD, periodontitis, Coexistence of CD and periodontal disease, and healthy controls were analyzed using 16 S rRNA sequencing to investigate the potential relationship between periodontitis and CD mediated by periodontal pathogens. In addition, the role of periodontal pathogens in the development of CD was further investigated via animal experiments.

## Methods

### Study population and sample collection

We synthesized information from 46 patients, including 12 patients diagnosed with Coexistence of CD and periodontal disease (Cp group), 12 patients diagnosed with periodontitis (Ps group), 10 patients diagnosed with CD (Ch group), and 12 healthy individuals (H group), enrolled from April 2018 to June 2019 at the General Hospital of Eastern Theater Command, Nanjing, China. The oral hygiene status of all teeth was assessed, and periodontal examinations were performed using CPI periodontal probes. Panoramic radiographs were also obtained from patients with periodontitis. The periodontal indices used included: bleeding on probing (BOP), probing depth, and clinical attachment loss (CAL). Patients were also examined for decay, missing, and filling teeth (DMFT). The dental examination was performed by two dentists. Each tooth is routinely examined at six sites: the distal, central, and mesial aspects of the buccal side, as well as the distal, central, and mesial aspects of the lingual side. Periodontitis was diagnosed according to the 2018 International New Classification of Periodontal Disease and Peri-Implant disease criteria [18]. The diagnostic criteria for CD were based on the Lennard–Jones criteria and were pathologically confirmed. The inclusion criteria for this study were as follows: (1) age 20–45 years; (2) persistent or recurrent diarrhea and abdominal pain, with a duration of at least six weeks; (3) positive CD results in stool routine examination and culture occurring at least thrice; (4) diffuse bleeding and ulceration in the rectal area revealed by colonoscopy; (5) pathological examination of mucosal biopsy specimens confirmed by CD diagnosis; (6) patients with CD having clinical diagnosis of periodontitis stage II (Cp group); (7) patients with CD having periodontal health confirmed by oral examination (Ch group); (8) patients diagnosed with periodontitis, based on the criteria in the consensus report from Workgroup 2 of the 2017 World Workshop on the Classification of Periodontal and Peri-implant Diseases and Conditions, and without CD (Ps group). H group matched the other three groups in sex and age, had no CD, had DMFT index ≤ 2 in oral examination, and was diagnosed with periodontal health. Periodontal health was defined by the absence of significant gingival inflammation (Bleeding on Probing (BOP) < 10%) and the lack of deep periodontal pockets and CAL on interproximal surfaces. The exclusion criteria were as follows: (1) patients with acute infectious enteritis, intestinal tuberculosis, colorectal cancer, Behcet’s disease, or other intestinal diseases; (2) patients who had undergone small intestine transplantation, fecal microbiota transplantation, colorectal resection, or other surgeries; (3) patients with diabetes, rheumatoid arthritis, malignant tumors, mental disorders, or other systemic diseases; (4) patients who had received intravenous or oral antibiotics in the past month; (5) patients who had used systemic corticosteroids, such as prednisone, biological agents, such as infliximab monoclonal antibody, immunosuppressants, such as salidurin, in the past three months; (6) smokers; (7) pregnant women or women taking contraceptives; (8) patients who were obese, with a body mass index ≥ 30; (9) patients who had received periodontal basic treatment or periodontal surgery in the past six months; (10) patients who were receiving orthodontic treatment or other oral treatments; (11) patients who were diagnosed with aggressive periodontitis according to the 1999 periodontal disease classification criteria or classified as grade C according to the 2018 new periodontal disease diagnosis criteria; (12) patients who could not cooperate with the physician for oral examination; (13) patients who refused to participate in this study. Individuals in the four groups were matched for sex and age. Written informed consent was obtained from all the participants before their participation in the study, which was approved by the Ethics Committee of the Eastern Theater Command General Hospital (NZGKJ-077, 2019). All the study procedures were performed in accordance with the Declaration of Helsinki.

### Saliva collection

The collection method was based on standard procedures of the Human Microbiology Project [19, 20]. Sampling was performed between 9:00–11:00 am. The patients avoided alcohol consumption, smoking, eating, and oral hygiene activities (including rinsing with mouthwash) for at least 1 h prior to sampling. 15 ml sterile centrifuge tubes were used to collect unstimulated saliva from the floor of the mouth, and the volume of each sample was about 2-3 ml. Unstimulated saliva samples were transferred to an ice box for storage in a -80 °C refrigerator for examination.

### 16 S rRNA sequencing

Total DNA was extracted using the QIAamp DNA Mini kit and purified using the Agencourt AMPure XP kit, according to the manufacturer’s protocol. The resulting DNA was stored in a -20 °C refrigerator after quality control by NanoDrop spectrophotometer. Bacterial 16 S rRNA V4 region was selected for amplification of the target gene with the primers (Beijing Ovison Gene Technology Co., Ltd.): 515 F (5’-GTGCCAGCMGCCGCGGTAA-3’); 806R (5’-GGACTACHVGGGTWTCTAAT-3’). PCR products were detected by 1% agarose gel electrophoresis and purified by automated purification using a magnetic bead method. MiSeq libraries were constructed and sequenced using the Illumina MiSeq platform (250 bp pair-end reads).

Data were processed using QIIME (v1.8.0) software. Sequence information was clustered into operational taxonomic units (OTU) for species classification according to barcode categorization, and the similarity was set to 97%. Species classification information corresponding to each OTU was obtained by comparison with the Silva database. Alpha and beta diversity analyses were performed using the Mothur software (version 1.31.2). Weighted UniFrace distance was used for cluster analysis using a pheatmap with the R (v3.1.1) software package.

### Animals

Male C57BL/6 mice aged 6–7 weeks were housed in a specific-pathogen-free environment of the Animal Experimental Center of Nanjing Medical University. They were kept under a 12-h light/dark cycle and a temperature of 20 to 22 °C, with free access to food and water. Animal experiments were conducted in accordance with the ARRIVE guidelines, approved by the Ethics Committee of Nanjing Medical University (license number: IACUC-2,102,014), and according to the Nanjing Medical University Health Guidelines for the Care and Use of Laboratory Animals.

### Dextran sulfate sodium (DSS)-induced colitis model

Mice received 3.0% (w/v) DSS (Sigma-Aldrich, UK) in sterile drinking water, administered continuously for seven days [21]. Subsequently, they received normal tap water for a three-day recovery period. The disease activity index was used to measure the severity of colitis [22] and was scored as body weight loss (0, none; 1, 1–5%; 2, 5–10%; 3, 10–20%; 4, > 20%), stool consistency (2, loose stools; 4, diarrhea), and bleeding (2, positive hemoccult; 4, gross bleeding).

### Bacterial strain intervention in mice

*Porphyromonas gingivalis* (*P. gingivalis*, ATTC W83, Baosai Biotechnology Co., Ltd, China) was resuscitated on a solid medium of Wilkins–Chalgren anaerobic broth (Oxoid, UK) containing 5% defibrinated sheep blood and 1.5–2.0% agar. After the formation of monoclonal colonies, they were selected and cultured in Wilkins–Chalgren anaerobic broth. *Fusobacterium nucleatum* (*F. nucleatum*, ATCC 25,586, Baosai Biotechnology Co., Ltd, China) was cultured on Columbia blood plates containing 5% defibrinated sheep blood and placed in anaerobic workstations. All media were placed in anaerobic workstations (90% N_2_, 5% CO_2_, 5% H_2_; 37℃). After collection, the bacteria were washed twice with sterile phosphate buffered saline (PBS). The optical density (OD) was used to estimate the number of bacteria in the culture by measuring at 660 nm (*P. gingivalis*, OD_660_ = 0.11, equivalent to 5 × 10^7^ CFU/mL; *F. nucleatum*, OD_660_ = 1 was equivalent to 1 × 10^9^ CFU/mL). Equal amounts of the bacterial suspension were centrifuged at 3500×g for 20 min. The supernatant was discarded, and the bacterial particles were suspended in PBS containing 2% carboxymethyl cellulose for animal experiments.

After successful induction of colitis in seven-day DSS, mice were randomized into three groups: one group was administered *P. gingivalis* continuously for 7 days (*P. gingivalis* group, *n* = 6), another group received *F. nucleatum* for 7 days (*F. nucleatum* group, *n* = 6), and the third group was given saline for 7 days as a control (DSS group, *n* = 6). The sham group (*n* = 6) was fed saline instead of DSS for 7 days as a negative control, followed by 3 days of normal tap, and then another 7 days of saline administration. The bacteria were dripped into the mouth of mice at a concentration of 10^10^ CFUs/mL in 50 µL suspension every 12 h by pipette to simulate the way of human saliva intake [23], instead of conventional intragastric treatment, which could better simulate the state of periodontal pathogenic bacteria intake into the body. After seven days of intervention, fecal samples were collected, mice were euthanized, colons were collected, and their lengths were measured.

### Hematoxylin and eosin staining

Colons were fixed in 4% PFA. The colons were then embedded in paraffin, sectioned, and stained with hematoxylin and eosin. They were then cut using a freezing microtome (Leica CM1900, Germany) and stained with hematoxylin and eosin (Servicebio, China). Images with magnifications of 100x and 400x were acquired at high magnification using CaseViewer 2.2.0 software for the assessment of colon inflammation. Histological scores were determined by goblet cell depletion (presence = 1, absence = 0), severity of epithelial/crypt loss (score, 0–4), muscle thickening (normal = 1, moderate = 2, extensive = 3), and extent of inflammatory cell infiltration in the lamina propria (score, 0–4) as previously described [24]. The scores for each measurement were summed to calculate the overall histological score for each sample.

### Real-time quantitative polymerase chain reaction

*P. gingivalis* and *F. nucleatum* were quantified in fecal samples of mice after oral administration. Bacterial genomic DNA was extracted using a DNA Rapid Extraction and Purification Kit (TianGen, China). PCR amplification was performed using *F. nucleatum* and *P. gingivalis* target gene fragments as templates supplemented with SYBR® Premix Ex Taq™ II (Tli RNaseH Plus, USA) and primers. The primers used to amplify the *Porphyromonas gingivalis* strain KCOM3188 and the *rpsD* gene of *F. nucleatum* are described in Table 1. The PCR reaction was prepared in 50 µl volume consisting of 25 µl of 2×Taq MasterMix (CWBIO), 1 µl of each primer (10 µmol), 1 µl of template DNA and 22 µl of distilled water. The amplification conditions were 94 °C for 5 min pre-denaturation, 94 °C for 30 s, 55 °C for 30 s, 72 °C for 30 s for 30 cycles, and then 72 °C for 10 min extension. Relative quantification of the genes was performed by BiosystemsTM QuantStudioTM 5 real-time PCR system and the 2^−ΔΔct^ analysis.

**Table 1:** Sequence of primers

		Primer sequence (5’ to 3’)
*P. gingivalis*	F:	TGTAGATGACTGATGGTGAAAACC
	R:	ACGTCATCCACACCTTCCTC
*F. nucleatum*	F:	GCCTGTTTTGAAGAAGTGTAGAGC
	R:	CTAACCAAGCTGGTGGAGTT

### ELISA

Levels of inflammatory cytokines in serum from mice were measured using TNF-α Kit, IL-1β Kit, and IL-6 Kit (Novus, USA) according to the manufacturer’s instructions. The absorbance at 450 nm was measured using a microplate reader (Multiskan™ GO; Thermo Fisher).

### Cell culture

Human colon cancer cells Caco-2 (Zhejiang Mason Cell Technology Co., Ltd., China) were cultured in Dulbecco’s modified Eagle’s medium (Gibco, USA), containing 20% fetal bovine serum (Clark, Australia), 1% penicillin-streptomycin, and 1% non-essential amino acids, and were maintained at 37 °C in a humidified chamber of 5% CO_2_. For monolayer differentiation and formation of tight junctions, the cells were removed enzymatically with 0.25% trypsin-EDTA, and cell suspension was seeded onto a 24-well plate. The medium was changed every other day. A total of 0.5 mL of the strains at a concentration of 1.0 × 10^8^ CFUs/mL in DMEM medium was added per well and incubated at 37 °C for 4 h (Barnett, Roy, Cookson, & McNabb, 2018). Cellular activity was determined using a Cell Counting Kit-8 (Beyotime).

### Immunofluorescence staining

Caco-2 cells in 24-well plates were cultivated with *F. nucleatum* and *P. gingivalis* (1.0 × 10^8^ cfu/mL) for 4 h. Caco-2 cells were washed once in PBS and fixed in 4% paraformaldehyde for 15 min at room temperature. Following three further PBS washes (5 min each time), cells were permeabilized in 0.5% Triton X-100 (Sigma-Aldrich, USA) and then incubated with a blocking buffer (5% donkey serum and 0.1% Triton X-100 in PBS) for 30 min. Primary antibody ZO-1 (1:100; Abcam) was diluted in PBS and incubated with the cells overnight at 4 °C. After three washes with PBST, Alexa Fluor-coupled secondary antibodies (Abcam, Cambridge, UK) were diluted 1:1000 and incubated for 1 h at room temperature. After three washes with PBST, the nuclei were counterstained with DAPI. Fluorescence was detected at the excitation wavelength of 488 nm and the emission wavelength of 550 nm using microplate reader (Multiskan™ GO; Thermo Fisher).

### Statistical analysis

Data were analyzed using the statistical software package SPSS 18.0 (SPSS Inc., Chicago, IL, USA). The Kolmogorov-Smirnov test was used to test the normality of continuous variables. For normally distributed continuous variables, the data were presented as mean ± standard deviation (SD), and differences between groups were evaluated using Analysis of Variance (ANOVA). In cases where continuous variables did not follow a normal distribution, the data were expressed as median (first quartile, third quartile), and the Kruskal-Wallis H test was employed to compare differences between groups. A *p-*value < 0.05 was considered statistically significant. All in vivo and in vitro experiments were independently repeated at least thrice.

## Results

### Clinical characteristics

The demographic data and clinical parameters of the study participants are shown in Table 2. There were also no significant differences in DMFT, BOP, periodontal pocket depth, CAL, and periodontitis severity (stage II) between patients in the Cp and Ps group, suggesting comparable levels of periodontitis progression. Similarly, the Harvey–Bradshaw index, used to assess CD status, also indicated no difference in the degree of intestinal inflammation between the Cp and Ch groups.

**Table 2:** Clinical indicators of the four study groups

	Cp (*n* = 12)	Ps (*n* = 12)	Ch (*n* = 10)	H (*n* = 12)	*P* value
Age (years)	36.5	36.9	36.4	34.3	0.367
Male (*n*)	7	7	5	7	/
Female (*n*)	5	5	5	5	/
DMFT	3.8 ± 1.6	4.3 ± 1.8	3.1 ± 1.5	1.2 ± 0.7	0.728
BOP %	45.1 (37.2, 61.6)	43.5 (27.8, 57.3)	4.4 (1.7, 6.7)	3.1 (1.2, 6.1)	0.188
PPD (mm)	3.7 ± 1.5	4.2 ± 2.3	1.9 ± 1.1	1.1 ± 0.9	0.29
CAL (mm)	2.9 ± 1.3	3.0 ± 1.9	0.5 ± 0.5	0.8 ± 0.5	0.27
Harvey-Bradshaw index	7 ± 1.5	/	6.5 ± 1.5	/	0.722

DMFT: missing or filled permanent teeth; BOP: bleeding on probing; PPD: periodontal pocket depth; CAL: clinical attachment loss; Cp: Coexistence of CD and periodontal disease; Ps: periodontitis; Ch: CD; H: healthy individuals. Each value is a mean ± SD.

### Bacterial characteristics of saliva samples in patients with CD and periodontitis

**Fig. 1:**
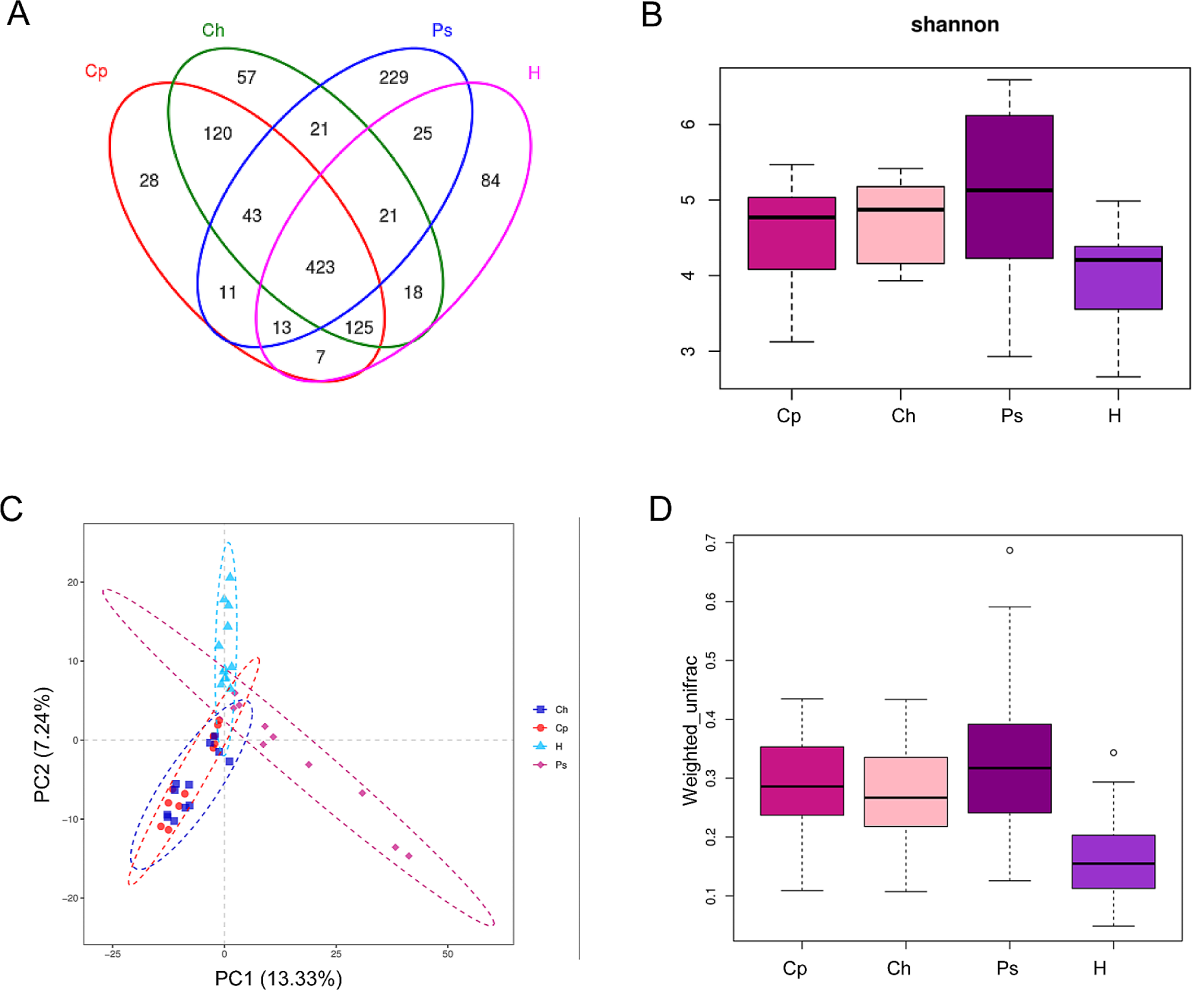
OTUs and microbial diversity analysis of saliva from patients with CD and/or periodontitis. **(A)** Venn diagram of the common and specific OTUs in saliva. **(B)** Alpha diversity of Shannon indexes between the sample types. **(C)** Least-squares discriminant analysis (PLS-DA) of UniFrac distances based on OTU composition and abundances. **(D)** Weighted UniFrac distance of β diversity. Cp, patients with CD and periodontitis; Ch, patients with CD; Ps, patients with periodontitis; H, healthy individuals; OTUs, operational taxonomic units; CD, Crohn’s disease

Saliva samples were collected from these patients, and DNA was extracted and subjected to 16 S rRNA gene sequencing. The samples were sequenced using the Illumina MiSeq sequencing platform, and 3,971,466 Clean Tags were obtained. As shown in Fig. 1A, a total of 1225 OTUs were generated. The results showed that 711 OTUs were shared by the Cp and Ch groups, whereas 490 OTUs were shared by the Cp and Ps groups. Shannon index showed that the bacterial α diversity of the Cp, Ch, and Ps groups was significantly higher than that in the H group, and α diversity in the Cp and Ch groups was more similar (Fig. 1B). For β diversity, partial Least-squares discriminant analysis of UniFrac distances between samples showed that microbial distribution of the Ps group was close to that of the Ch group (Fig. 1C), while they were all separated from the other two groups. Weighted Unifrace distance also showed similar β diversity of the Cp and Ch groups (Fig. 1D). These results suggested that the bacterial diversity of the Cp group was more similar to that of the Ch group.

**Fig. 2:**
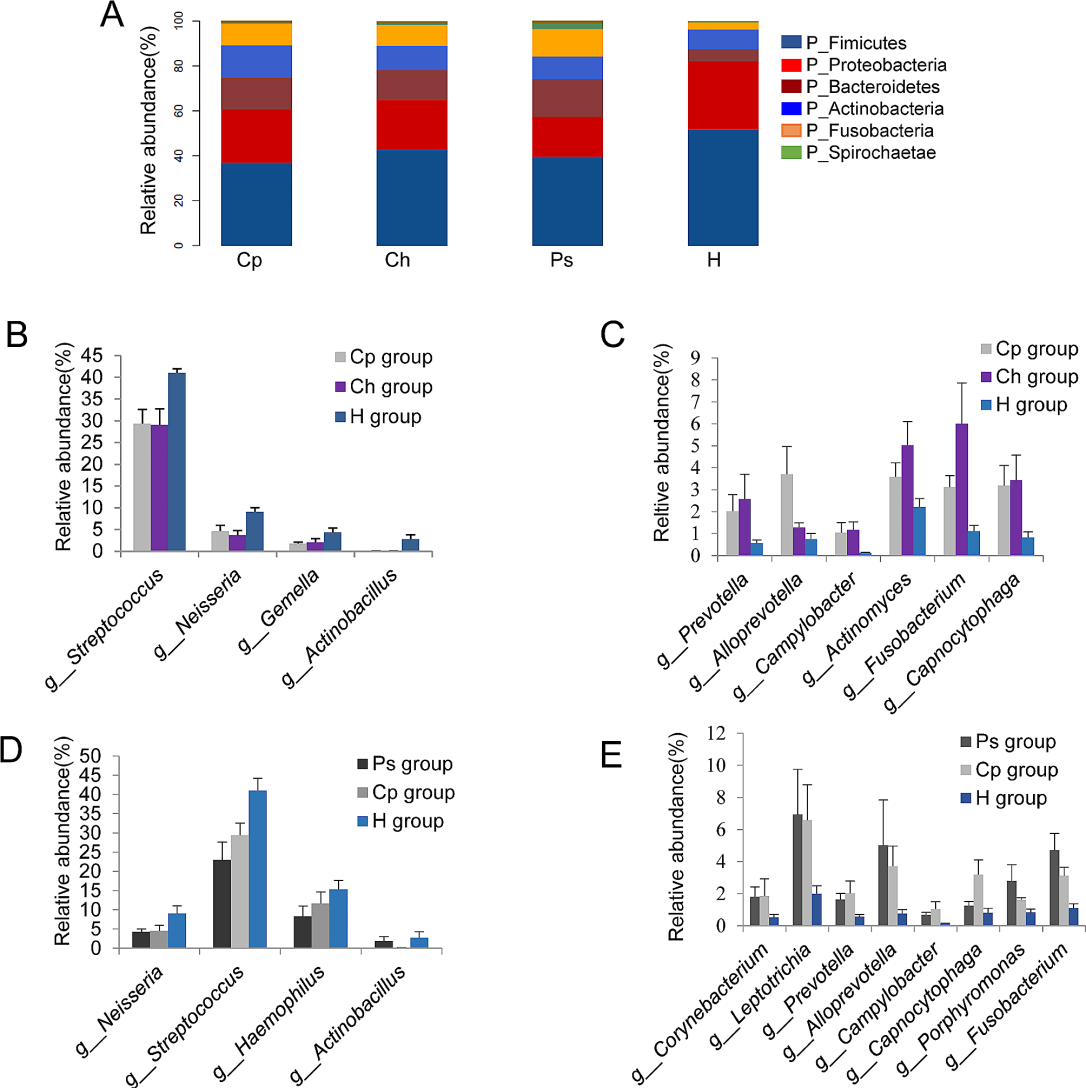
Microbial composition of saliva from patients with CD and/or periodontitis. **(A)** Microbial composition across all samples at the phylum level. **(B) (C)** Microbial composition across samples of Cp, Ch, and H groups at the genus level. **(D)** Microbial composition across samples of Cp, Ps, and H groups at the genus level. Cp, patients with CD and periodontitis; Ch, patients with CD; Ps, patients with periodontitis; H, healthy individuals; CD, Crohn’s disease

Compared with that in healthy controls, bacteria at the phylum level showed that the relative abundances of *Firmicutes* and *Proteobacteria* were significantly decreased, whereas the relative abundances of *Bacteroidetes, Fusobacterium*, and *Actinobacteria* were significantly increased in the Ps and Ch groups (Fig. 2A, *P* < 0.05). At the genus level, the Cp and Ch groups also showed similar changes compared with that in the healthy controls, including significant decreases in *Streptococcus, Neisseria, Gemella*, and *Actinobacillus* (Fig. 2B, *P* < 0.05) and a significant increase in *Prevotella, Alloprevotella, Campylobacter, Actinomyces, Fusobacterium*, and *Capnocytophaga* (Fig. 2C, *P* < 0.05). The relative abundances of *Streptococcus, Neisseria, Haemophilus*, and *Actinobacillus* in the Ps and Cp groups were significantly lower than those in the H group (Fig. 2D, *P* < 0.05), whereas those of *Corynebacterium, Leptotrichia, Prevotella, Alloprevotella, Campylobacter, Capnocytophaga, Porphyromonas*, and *Fusobacterium* were significantly higher than those in the H group (Fig. 2E, *P* < 0.05). In general, changes in the oral microbiota in Coexistence of CD and periodontal disease are partially consistent with those in the oral microecology of CD, suggesting that disturbances in the oral microbiome caused by CD may contribute to the development of Coexistence of CD and periodontal disease.

**Fig. 3:**
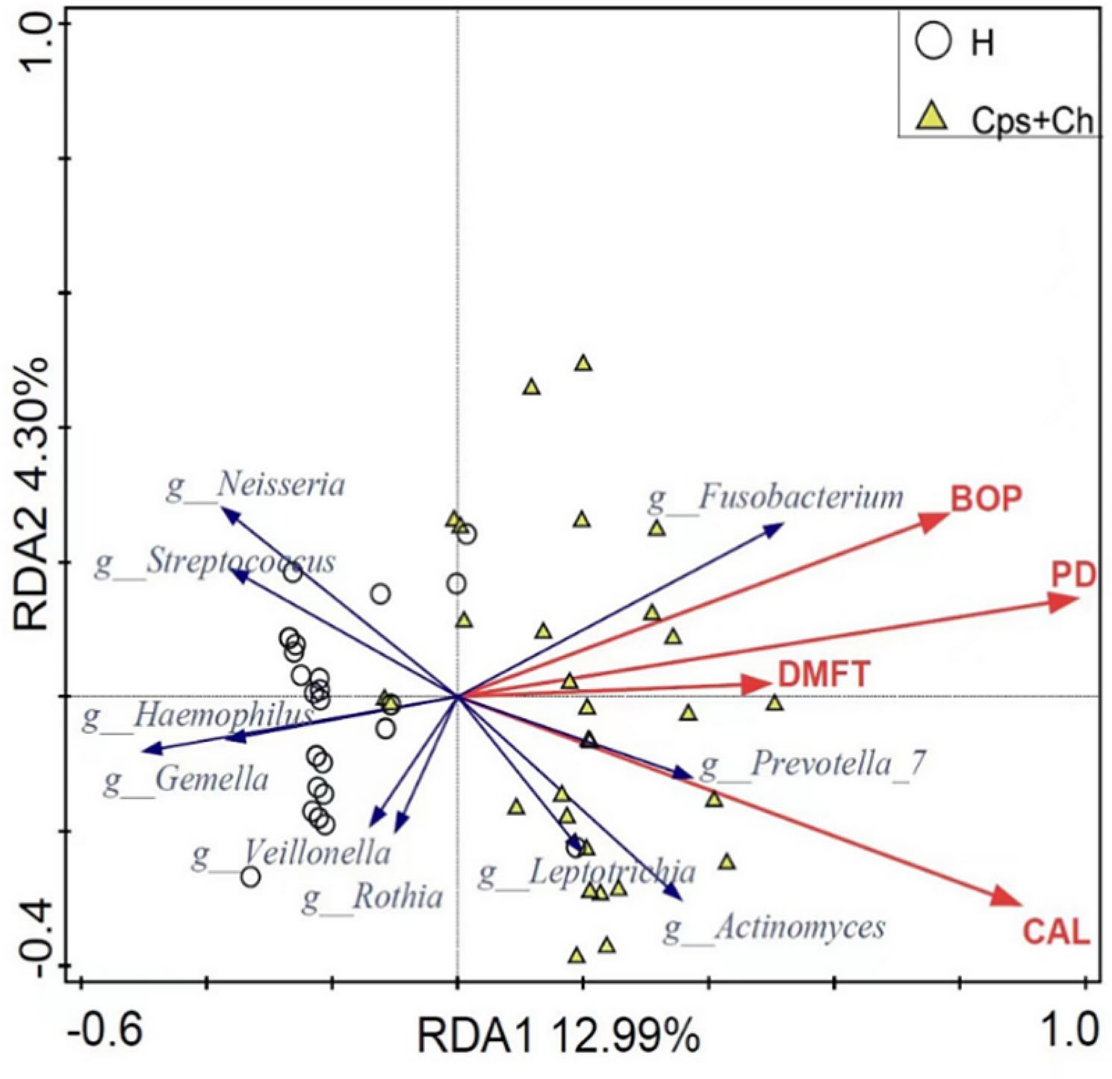
Correlation between periodontitis index and top ten bacterial genera in the Cp and Ch groups. DMFT, missing or filled permanent teeth; BOP, bleeding on probing; PPD, periodontal pocket depth; CAL, clinical attachment loss; Cp, patients with Crohn’s disease and periodontitis; Ch, patients with Crohn’s disease;

Correlation analysis was performed to further reveal the relationship between the oral microbial community and the clinical indicators of Coexistence of CD and periodontal disease, including DMFT, BOP, periodontal pocket depth, and CAL (Fig. 3). The results showed that the abundance of *Fusobacterium, Actinomyces*, and *Prevotella*, enriched in the Cp and Ch groups, were positively correlated with periodontitis indicators. In contrast, the abundance of *Streptococcus, Neisseria*, and *Gemella*, which decreased in the Cp and Ch groups, was negatively correlated with the severity of periodontitis. These results indicate that the occurrence of periodontitis in CD is closely related to disorders of the oral microbial structure in CD. In general, the presence of periodontal pathogens in CD can further aggravate Coexistence of CD and periodontal disease.

### *P. gingivalis* and *F. nucleatum* accelerated CD progression induced by DSS in a mouse model

To further demonstrate the relationship between periodontitis and CD, key periodontal pathogens were used in a mouse model. *F. nucleatum*, belonging to *Fusobacterium* that was enriched in Cp and played an important role in adhesion and copolymerization with other pathogens, resulting in destruction of periodontal tissues [25]. *P. gingivalis*, belonging to the genus *Porphyromonas*, was enriched in Cp; it is an important pathogen involved in periodontitis [26]. These were selected as key periodontal pathogens to explore their roles in the development of CD.

**Fig. 4:**
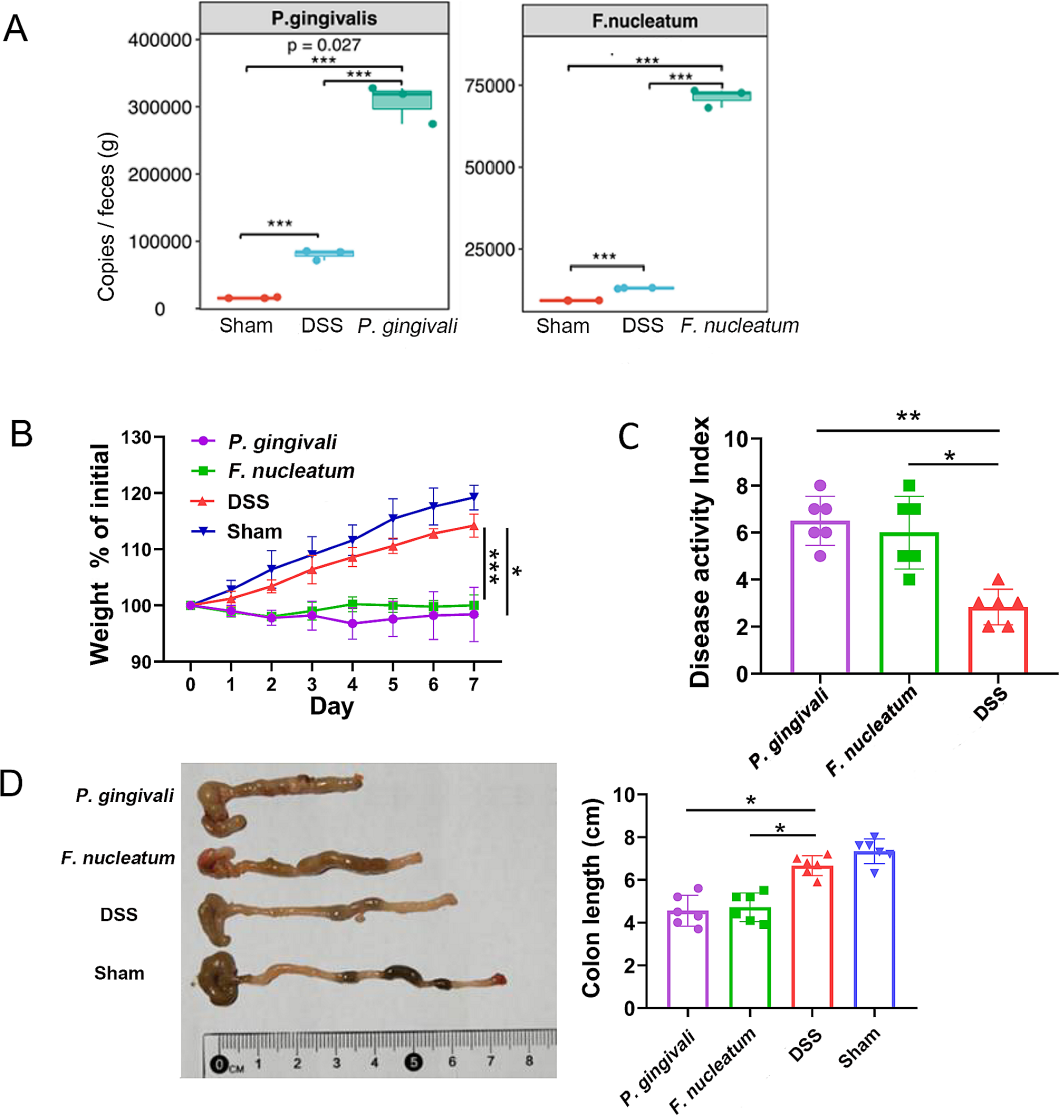
Periodontal pathogens accelerated the progression of DSS-induced CD in a mouse model. **(A)** Average content of periodontal pathogens in fecal samples of each group. **(B)** Weight loss (the day’s body weight/weight on day 0). **(C)** Disease activity index of CD mice after 7-day periodontal pathogens gavage treatment. **(D)** Colon length of mice at sacrifice. *Pg*, *Porphyromonas gingivalis; Fn, Fusobacterium nucleatum*; CD, Crohn’s disease; DSS, dextran sulfate sodium

First, *P. gingivalis* and *F. nucleatum* were quantified in fecal samples of mice after oral administration. As shown in Fig. 4A, the specific bacterial load in the *P. gingivalis* and *F. nucleatum* groups was significantly higher than that in the DSS-saline group, indicating that *P. gingivalis* and *F. nucleatum* administered orally could stably colonize the gut. Subsequently, we found that the *P. gingivalis* and *F. nucleatum* groups showed significant weight loss (Fig. 4B, *P* < 0.05) and higher disease activity index scores (Fig. 4C). Additionally, *P. gingivalis* and *F. nucleatum* decreased the colon length in DSS-pretreated mice (Fig. 4D, *P* < 0.05), indicating that intestinal inflammation persisted and was gradually aggravated after periodontal pathogen intervention.

### Periodontal pathogens exacerbated intestinal and systemic inflammation

**Fig. 5:**
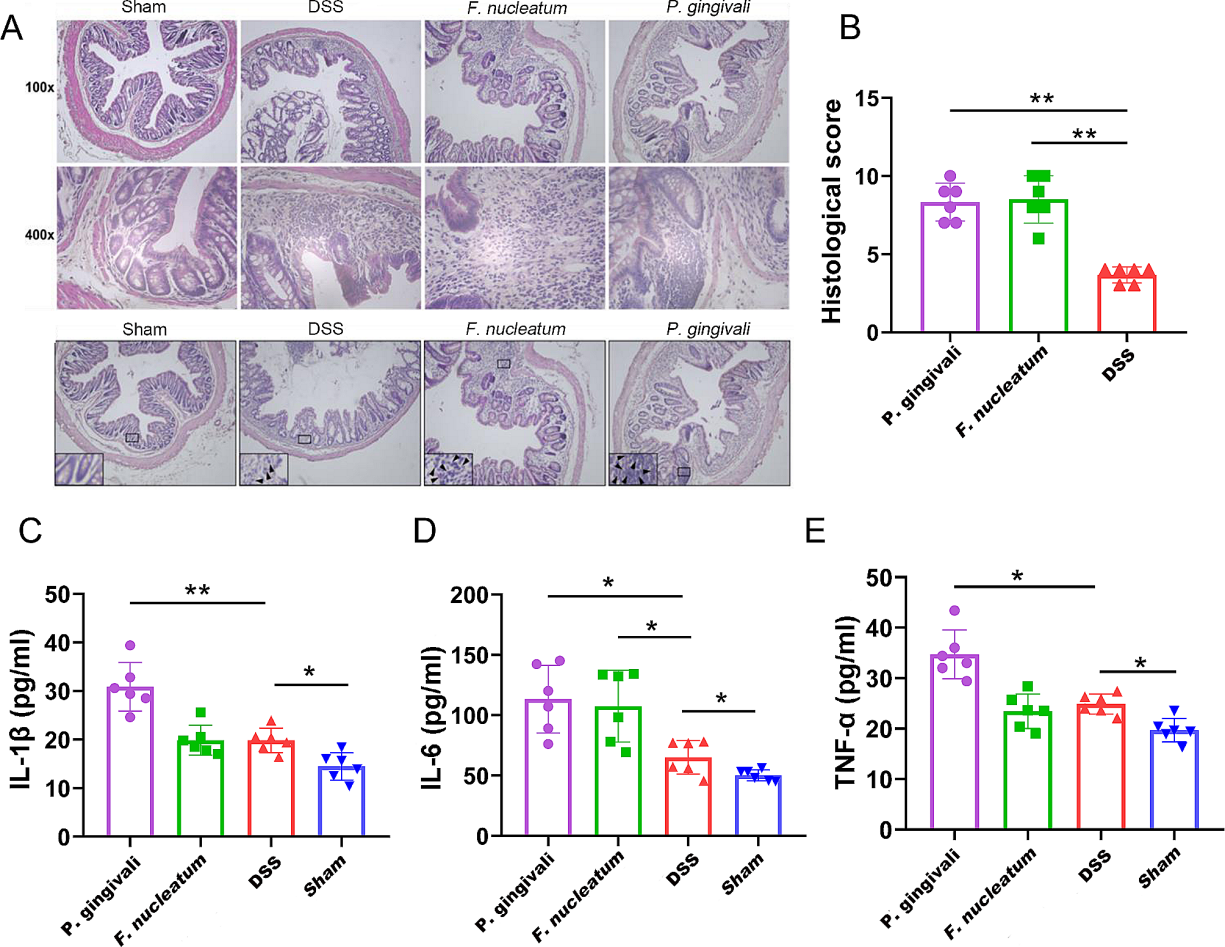
Periodontal pathogens exacerbated intestinal and systemic inflammation. **(A)** Hematoxylin and eosin staining of colon in each group. **(B)** Histological score for colitis of each CD sample. Levels of serum inflammatory cytokines IL-1β **(C)**, IL-6 **(D)**, and TNF-α **(E)** in each group. CD, Crohn’s disease

The severity of colonic inflammation and ulceration was assessed via histological examination. It showed distinct infiltration of inflammatory cells, loss of crypts, and destruction of the mucosal layer in *P. gingivalis* and *F. nucleatum* administered mice (Fig. 5A), whereas mice discontinued with DSS showed only mild inflammation. Similarly, the proinflammatory effects of *P. gingivalis* and *F. nucleatum* were observed in the histological score (Fig. 5B; all, *P* < 0.01). After *P. gingivalis* administration, IL-1β (Fig. 5C, *P* < 0.01), IL-6 (Fig. 5D, *P* < 0.05), and TNF-α (Fig. 5E, *P* < 0.05) levels were significantly higher than those in the DSS group. IL-6 was also significantly elevated in the *F. nucleatum* group (Fig. 5D, *P* < 0.05) while no significant differences were observed in IL-1β and TNF-α levels. These results suggest that periodontal pathogens can lead to further deterioration of intestinal and systemic inflammation.

### Effect of periodontal pathogens on intestinal epithelial cells in vitro

**Fig. 6:**
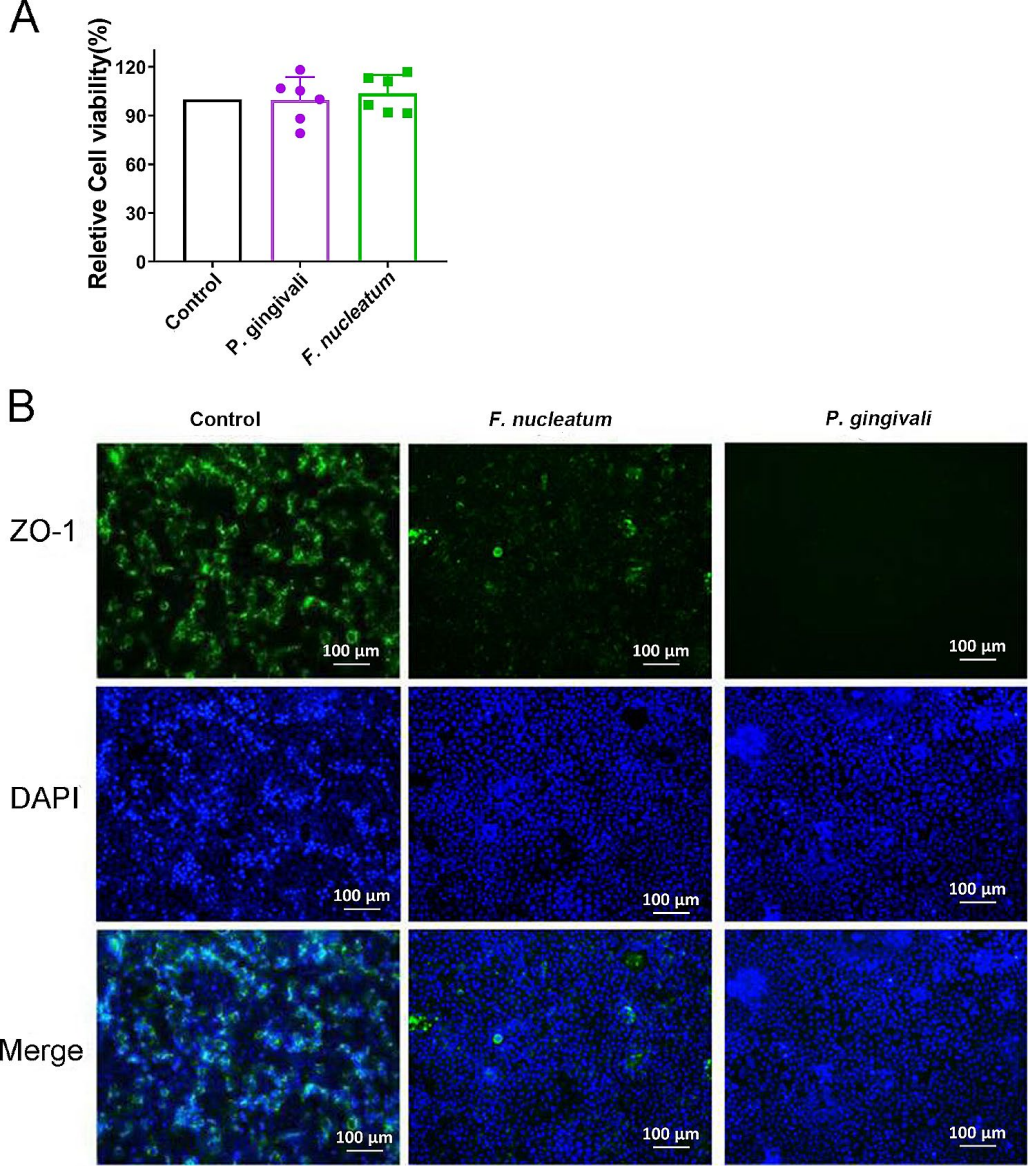
Caco-2/periodontal pathogen coculture in vitro. Cellular activity determined using the Cell Counting Kit-8. Immunofluorescence staining of tight junction protein zonula occludens protein 1 (ZO-1, green) in Caco-2 cells

Periodontal pathogens can migrate to the gut and produce their effects by directly contacting cells. We used a Caco-2/periodontal pathogen coculture in vitro to further understand the mechanism by which periodontal pathogens affect intestinal inflammation. First, cell viability assays demonstrated that neither *P. gingivalis* nor *F. nucleatum* affected the viability of Caco-2 cells (Fig. 6A). Immunofluorescence staining showed that the green fluorescence of the *P. gingivalis* and *F. nucleatum* groups almost completely disappeared after 4 h of coculture (Fig. 6B), indicating that the tight junction protein ZO-1 in the intestinal epithelial cells was lysed. This demonstrates that periodontal pathogens can increase intestinal permeability by directly disrupting tight junctions of the intestinal epithelium.

## Discussion

The link between CD and periodontitis has been extensively researched [15, 27], and the evidence strongly suggests that there is a significant association between the two conditions. Patients with CD are more likely to develop periodontitis, and the disease tends to be more severe in these individuals [28]. Periodontitis has also been found to worsen symptoms [29]. Although the exact mechanisms underlying this association remain unclear, it is thought that changes in microbiota and pathogen invasion can disrupt microecological homeostasis, leading to an imbalance that further exacerbates the disease [30]. Oral microorganisms can be transmitted to the gastrointestinal tract [31], providing a basis for oral pathogens to disrupt the gut homeostasis. Therefore, disruption of oral microbiota may be an important mediator in the relationship between CD and periodontitis.

16 S rRNA sequencing revealed an imbalance in the oral microbiota of patients with Coexistence of CD and periodontal disease. Among the pathogenic bacteria identified, *Corynebacterium* encodes genes for virulence and multidrug resistance [32], while *Leptotrichia* has been reported as a potential pathogen that can spread into the bloodstream when the mucosal barrier is compromised [33]. An increased abundance of *Prevotella* has been linked to mucosal inflammation mediated by helper type 17 T cells [34], while *Alloprevotella* is an obligatory anaerobic bacillus associated with oral dysbiotic infections [35]. *Capnocytophaga* is known to cause local and systemic infections [36]. These pathogens are significantly enriched in the oral cavity of patients with periodontitis and Coexistence of CD and periodontal disease, indicating their pathogenic role in CD. Additionally, *Capnocytophaga, Prevotella, Alloprevotella, Campylobacter, Fusobacterium, Streptococcus, Neisseria*, and *Actinobacillus* consistently changed in the oral cavity of CD, Coexistence of CD and periodontal disease and periodontitis, and may serve as potential biomarkers for identifying the “gum–gut axis.”

Pathogens in the oral cavity of patients with CD may promote the development of periodontitis. The opportunistic pathogens *Fusobacteria* and *Prevotella*, which are enriched in the oral cavity of patients with periodontitis and CD, were positively correlated with the clinical indicators of periodontitis, including BOP, PD, and CAL. Conversely, significantly decreased numbers of pathogens in the oral cavity, such as *Streptococcus*, *Neisseria*, *Haemophilus*, and *Gemella*, were negatively correlated with the severity of periodontitis. This suggests that an ecological imbalance in the oral microbiota is the underlying cause of susceptibility to periodontitis in patients with CD.

To further support the idea that periodontal pathogens can migrate from the oral cavity to the intestine and contribute to the development of intestinal diseases, we focused on two key pathogens involved in periodontitis: *F. nucleatum* and *P. gingivalis*. *F. nucleatum* is a periodontal pathogen significantly enriched in the oral cavity of patients with periodontitis. Studies have shown that *F. nucleatum* migrates from major colonizing sites in the oral cavity to the intestines [37]. Additionally, it can interact with other pathogens and generate local inflammatory microenvironments, accelerating the progression of periodontitis [38]. Similarly, *P. gingivalis* is also considered a critical pathogen in periodontitis [39] and a risk factor for intestinal inflammation owing to its ability to alter microbiota composition and metabolite profiles [40]. Although mice in the DSS group did not receive any *P. gingivalis* or *F. nucleatum* inoculation during the entire modeling period, both bacteria were positive, which indirectly indicated that intestinal inflammation was related to the translocation of periodontal pathogens. This result is also consistent with the relevant reports on IBD [41, 42].

Following intervention with periodontal pathogens, the disease index of CD in mice increased and intestinal inflammation was exacerbated. *F. nucleatum* can secrete several harmful molecules in the intestine, which can alter microbe-host interactions, causing damage to the epithelium in CD [43]. *P. gingivalis* has various toxic components, such as gingipains and lipopolysaccharides, which adhere to the intestinal epithelium through the capsule and pili and evade the host immune response, leading to local inflammation [44]. In addition, circulating levels of proinflammatory cytokines IL-1β, IL-6, and TNF-α were increased in CD mice, indicating that proinflammatory cytokines could be released into the blood through damaged intestinal tissues, causing systemic inflammation. In periodontal pathogen/Caco-2 coculture, pathogens can directly disrupt the expression of the tight junction protein ZO-1 in intestinal cells. ZO-1 is a major transmembrane tight junction protein that is critical for regulating intestinal barrier function [45]. Periodontal pathogens can colonize and translocate into the intestine, leading to local intestinal inflammation. Additionally, periodontal pathogens can directly act on the intestinal epithelium and increase the permeability of the intestinal barrier by affecting the expression of intestinal tight junction proteins, thereby amplifying the inflammatory response and promoting CD development through the release of proinflammatory cytokines.

Overall, our study provides compelling evidence of structural disorders in the oral microecology of patients with CD, which are closely linked to the development of periodontitis, as demonstrated by microbial detection and analysis. Moreover, we showed that key periodontal pathogens have the potential to be pathogenic to inflammatory bowel through both direct and indirect pathways, contributing to the development of CD. This study elucidated the potential mechanisms underlying the relationship between periodontitis and CD and identified key pathogenic microorganisms that may promote CD-related periodontitis progression. This study sheds light on the complex interplay between oral microbiota and intestinal disease, paving the way for the development of novel diagnostic tools and therapeutic strategies targeting specific microbial populations. Although our study explored the relationship between CD and periodontitis in human, animal, and cellular models from a microbiological perspective, unresolved scientific problems remain. The diversity of periodontal pathogens and their synergistic effects make the selection of unique representative pathogenic strains challenging. Moving forward, we are committed to further investigating the specific functions and balance mechanisms of the microbiota based on toxic molecules and pathways to gain a deeper understanding of this complex biological interplay.

## Conclusion

The structural disturbance of oral microecology in patients with CD is closely related to the development of periodontitis, and animal experiments support the idea that these pathogens can migrate to the intestine and contribute to intestinal diseases.

### Electronic supplementary material

Below is the link to the electronic supplementary material.

Supplementary Material 1

## Data Availability

Data supporting this study are available from the corresponding author upon reasonable request. All the raw sequences have been stored in the NCBI Sequence Read Archive (SRA) with the login number PRJNA949975.

## Abbreviations

CD: Crohn’s disease
*P. gingivalis*: *Porphyromonas gingivalis*
*F. nucleatum*: *Fusobacterium nucleatum*
IBD: Inflammatory bowel disease
PBS: Phosphate buffered saline
OD: Optical density
ANOVA: Analysis of variance
PLS-DA: Partial Least-squares discriminant analysis
