# Supplementary figures and images for: Key periodontal pathogens may mediate potential pathogenic relationships between periodontitis and crohn’s disease
Source: BMC Oral Health. 2024 Jun 7;24:668. doi: 10.1186/s12903-024-04425-0 (PMC11161938; doi:10.1186/s12903-024-04425-0)

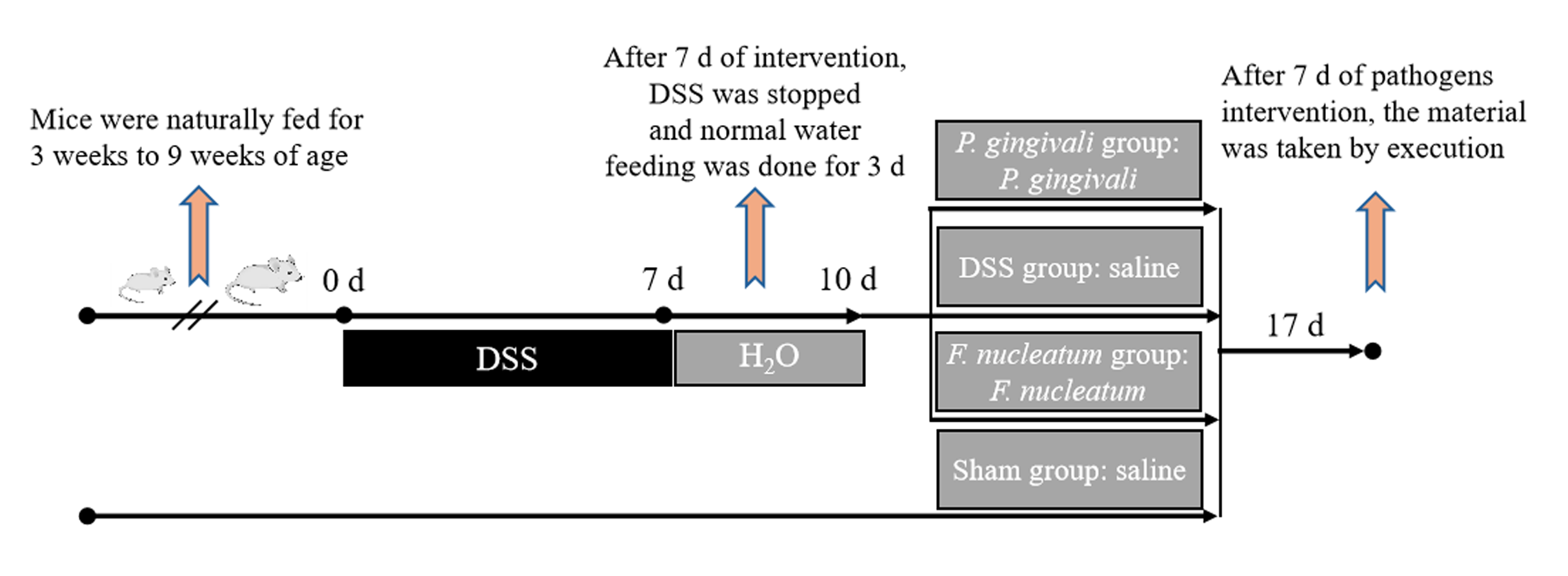

Supplement: Supplementary file 1 — Supplementary Material 1 [file 12903_2024_4425_MOESM1_ESM.tif]
